# Field courses narrow demographic achievement gaps in ecology and evolutionary biology

**DOI:** 10.1002/ece3.6300

**Published:** 2020-05-08

**Authors:** Roxanne S. Beltran, Erin Marnocha, Alexandra Race, Donald A. Croll, Gage H. Dayton, Erika S. Zavaleta

**Affiliations:** ^1^ Ecology and Evolutionary Biology University of California Santa Cruz CA USA; ^2^ Natural Reserve System University of California Oakland CA USA; ^3^ Education University of California Santa Cruz CA USA; ^4^ Natural Reserve System University of California Santa Cruz CA USA

**Keywords:** assessments, marginalized, minority, outcomes, STEM, student, success

## Abstract

Disparities remain in the representation of marginalized students in STEM. Classroom‐based experiential learning opportunities can increase student confidence and academic success; however, the effectiveness of extending learning to outdoor settings is unknown. Our objectives were to examine (a) demographic gaps in ecology and evolutionary biology (EEB) major completion, college graduation, and GPAs for students who did and did not enroll in field courses, (b) whether under‐represented demographic groups were less likely to enroll in field courses, and (c) whether under‐represented demographic groups were more likely to feel increased competency in science‐related tasks (hereafter, self‐efficacy) after participating in field courses. We compared the relationships among academic success measures and demographic data (race/ethnicity, socioeconomic status, first‐generation, and gender) for UC Santa Cruz undergraduate students admitted between 2008 and 2019 who participated in field courses (*N* = 941 students) and who did not (*N* = 28,215 students). Additionally, we administered longitudinal surveys to evaluate self‐efficacy gains during field‐based versus classroom‐based courses (*N* = 570 students). We found no differences in the proportion of students matriculating at the university as undecided, proposed EEB, or proposed other majors across demographic groups. However, five years later, under‐represented students were significantly less likely to graduate with EEB degrees, indicating retention rather than recruitment drives disparities in representation. This retention gap is partly due to a lower rate of college completion and partly through attrition to other majors. Although under‐represented students were less likely to enroll in field courses, field courses were associated with higher self‐efficacy gains, higher college graduation rates, higher EEB major retention, and higher GPAs at graduation. All demographic groups experienced significant increases in self‐efficacy during field‐based but not lecture‐based courses. Together, our findings suggest that increasing the number of field courses and actively facilitating access to students from under‐represented groups can be a powerful tool for increasing STEM diversity.

## Graphical abstract

We examined the relationships among academic success measures, field course enrollment, and diversity metrics (race/ethnicity, socioeconomic status, first‐generation, and gender) for 28,500 undergraduate students admitted to the University of California between 2008 and 2014. We found that field courses increase self‐confidence, graduation rates, and retention of under‐represented students in ecology fields.

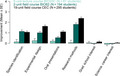

## 1 INTRODUCTION

Gender and racial representation disparities remain a concerning issue in Science, Technology, Engineering, and Math (STEM) fields worldwide (Holman, Stuart‐Fox, & Hauser, 2018). The leaky pipeline begins with high attrition of students (Graham, Frederick, Byars‐Winston, Hunter, & Handelsman, 2013), especially racial/ethnic minority, economically disadvantaged, first‐generation, and women students (Alexander, Chen, & Grumbach, 2009; Reardon, 2011; Riegle‐Crumb, King, & Irizarry, 2019). College has been identified as a critical time point in the recruitment and retention of diverse students in STEM.

Ensuring diversity in STEM requires examining both racial and gender identities as well as socioeconomic status and how these demographic factors intersect (Eaton, Saunders, Jacobson, & West, 2019). In some cases, student demographics are directly related to sociological, psychological, and physiological mechanisms such as test anxiety (Salehi et al., 2019) that in turn compromise academic performance (Ballen, Wieman, Salehi, Searle, & Zamudio, 2017) and alter career intentions (Cheryan, Ziegler, Montoya, & Jiang, 2017; Epstein & Fischer, 2017). As a result, targeted interventions such as diversity programs and workshops have been created to combat social isolation and low confidence (Ahern‐Dodson, Clark, Mourad, & Reynolds, 2020; Ballen & Mason, 2017; Casad et al., 2018; Yeager et al., 2016). These efforts have shown some success in closing gaps in achievement metrics between marginalized students and their peers (Cohen, Garcia, Apfel, & Master, 2006). However, these interventions are costly and difficult to scale, and other research has shown that interventions in classroom and other course‐based learning environments may be preferable (see citations in Ballen et al. (2018)).

As a result, there has been an explosion of discipline‐based education research demonstrating that students can be supported through innovative approaches to active learning, course structure, and real‐time evaluations (Allen & Tanner, 2005; Canning, Muenks, Green, & Murphy, 2019; Cotner & Ballen, 2017; Freeman et al., 2014; Lovelace & Brickman, 2013; Owens et al., 2017; Seidel, Reggi, Schinske, Burrus, & Tanner, 2015; Sullivan, Ballen, & Cotner, 2018; Tanner, 2013; Weir et al., 2019). These innovative teaching methods have been shown to play a particularly important role for marginalized groups, such as those excluded based on gender (Freeman et al., 2007; Haak, HilleRisLambers, Pitre, & Freeman, 2011; Lorenzo, Crouch, & Mazur, 2006), race (Ballen et al., 2017), and financial status (Claro, Paunesku, & Dweck, 2016). Student‐driven inquiry is another high‐impact educational practice (Kuh, 2008) that has been implemented successfully in undergraduate classrooms (Ballen et al., 2017; Lopatto, 2007; Weaver, Russell, & Wink, 2008). Research opportunities have increased student confidence in the ability to do science (hereafter, self‐efficacy) and in turn help students develop their science identity and sense of belonging and increase motivation (Linnenbrink & Pintrich, 2003; Rodenbusch, Hernandez, Simmons, & Dolan, 2016; Schultz et al., 2011; Schunk, 1991; Walton & Cohen, 2011). These initiatives can also lead to publications and presentation opportunities that build confidence and experience (Ballen & Mason, 2017). While these course‐based undergraduate research experiences traditionally take place in laboratory settings (Adams, 2009), a growing number take place during short‐term field trips (Thompson, Neill, Wiederhoeft, & Cotner, 2016).

The success of extending teaching to outdoor settings has led to entire courses based around field‐based learning (hereafter, field courses) that facilitate hands‐on activities and inquiry‐based research. These courses focus on replacing a competitive model of learning with a team model of achievement in which collaboration and shared learning are valued. Thus, field‐based courses provide several potentially high‐impact services including deep immersion in the subject matter, active learning, formation of peer networks and cohesive learning communities (Epstein, Godsoe, & Kosinski‐Collins, 2015; Toven‐Lindsey, Levis‐Fitzgerald, Barber, & Hasson, 2015), enabling international field experiences (Bruening & Frick, 2004), having small class sizes that facilitate participation (Ballen et al., 2019), and allowing student‐faculty interactions that could lead to future opportunities (Kim & Sax, 2009; Newman, 2011; Wilson et al., 2012). These factors make field courses a promising potential tool for reducing attrition in STEM, especially the life sciences. Many research efforts have demonstrated the short‐term cognitive benefits of these courses (Boyle et al., 2007; Cotton, 2009; Easton & Gilburn, 2012), but none have evaluated long‐term benefits such as college completion, academic success, and retention in STEM. Further, it is unclear how these field courses might benefit undergraduate students from groups traditionally under‐represented in the sciences. Field courses may be especially beneficial for under‐represented students by reducing disparities in self‐efficacy and core science skills. That said, field courses are typically optional, costly, and selective, so it is also important to understand whether these potential barriers lead to disparities in field course participation across demographic groups.

The University of California Santa Cruz (UCSC) is an ideal setting to examine these questions. The campus offers extensive field‐based undergraduate science opportunities, including over 60 field courses that serve more than 2,000 students each year. UCSC is also a minority‐serving institution with over 38% under‐represented minority students, 45% first‐generation college students, and 40% from households with incomes <$50,000 (UC InfoCenter). Our objectives were to examine (a) demographic gaps in ecology and evolutionary biology (EEB) major completion, college graduation rates and grade point averages for students that did and did not enroll in field courses, (b) whether under‐represented demographic groups were less likely to enroll in field courses than their peers, and (c) whether under‐represented demographic groups were more likely to experience self‐efficacy gains from field course participation than their peers. Given that lecture‐based courses are traditionally used to fulfill major requirements, we compare enrollment metrics in objective 2 and self‐efficacy gains in objective 3 across lecture and field courses. We hypothesized that field course participation could close demographic self‐efficacy and achievement gaps in EEB by increasing self‐efficacy.

## 2 MATERIALS AND METHODS

This work was approved by UCSC IRB protocol #HS3230. For each student admitted to UCSC between Fall 2008 and Spring 2014 (*N* = 28,500), we obtained academic data and self‐reported demographic data from the registrar. Data were de‐identified to protect student identities. Demographic data were scored as binomial (Yes = 1, No = 0) for four categories: (a) URM, under‐represented minority status (students who identify primarily as African American/Black, American Indian/Alaskan Native, and Hispanic/Latino); (b) FIF, first‐in‐family to attend college; (c) EOP, whether they are part of the Educational Opportunity Program based on family income, undocumented, and foster care status); and (d) gender. Students who declined to state (*N* = 196, or 0.7%) and those who indicated nonbinary gender (*N* = 30, or 0.1%) were not included in the gender comparison analyses to avoid inadvertent disclosure of student identities. Students who did not wish to specify their characteristics were not included in the analysis. Academic data included proposed major at admission (proposed Ecology and Evolutionary Biology (EEB): Ecology & Evolution, Marine Biology, Plant Sciences, Biology, *N* = 2,057; proposed non‐EEB: *N* = 22,685, or undecided: *N* = 3,758), whether they graduated in five years, major at graduation, grade point average at graduation (hereafter, GPA), and number of field courses completed.

To analyze associations between student demographic categories, we calculated bivariate Spearman correlation coefficients. We present Cramer's V values, which provide information on association for nominal variables scaled between 0 (no association) and 1 (full association). To quantify how the demographics of field courses have changed over time, we extracted historical demographic composition data from field courses taught between 2008 and 2019 (*N* = 1,239 students; courses BIO75, BIO82, BIO128L, BIO151, BIO159, BIO161), and the gateway lecture course for EEB majors (*N* = 11,589 students; BIO20C). Field courses were included if more than 50% of course hours were spent in the field rather than in the classroom and if the course had been taught for at least 4 years. Information about course name, size, fee, and equipment requirements for each field course is provided in Appendix Table S1.

To quantify the association between field course participation, graduation rates, and retention in EEB, we constructed contingency tables of outcomes based on admission major and field course enrollment. We used chi‐square tests to evaluate the dependence of these factors, separated by admission major, and report odds ratios from the R package *epitools*. For students who graduated within five years, we used hierarchical linear regression models to quantify the relative contributions of admission and graduation major, student demographics, and field course enrollment on graduation GPA. We provide metrics on the overall variance explained by each model (R^2^) along with the unstandardized (B) and standardized (β) coefficients of each parameter within the models from R package *lm.beta*.

To evaluate self‐efficacy gains during field courses, we administered longitudinal surveys in a subset of three courses between Fall 2016 and Spring 2019: (a) BIOE 20C, a gateway ecology and evolutionary biology lecture course (*N* = 81); (b) BIOE 82, a 2‐unit, lower‐division field course that is intended to provide early field immersion and introduce natural history information and field research opportunities to students (*N* = 194); and (c) CEC, an immersive 19‐unit upper‐division field course that engages students in student‐directed research projects (*N* = 295). While BIOE 20C and BIOE 82 are courses offered at UCSC, CEC is a UC system‐wide course that enrolls students from all UC campuses. We administered paired pre‐ (first week of the academic quarter) and post‐ (last week of the academic quarter) surveys. Each student was asked to rate their confidence on a 5‐point Likert scale (1 = Strongly Disagree, 2 = Disagree, 3 = Neither Agree Nor Disagree, 4 = Agree, and 5 = Strongly Agree) for each of six questions: (a) I am familiar with the flora, fauna, and ecosystems of California; (b) I have strong experimental design skills; (c) I have strong oral presentation skills; (d) I know how to conduct field research projects from start to finish; (e) I am interested in pursuing a career in science; (f) I am interested in pursuing a graduate degree. We undertook two steps to validate the survey for cross‐group reliability. First, we used principal components analysis factor loading values to determine the number of common themes represented by the 6 difference (post–pre) survey questions. We identified two elements represented by survey questions: self‐efficacy (represented by questions 1–4) and motivation (represented by questions 5–6). Next, we calculated a Cronbach's alpha value as 0.7 [95% confidence boundaries 0.67 to 0.74] using the R package *psych* to measure the reliability of the survey (Cronbach, 1951). The improvement across the course (calculated as the difference between post‐ and pre‐surveys for each question) is presented. Data were aggregated by course and demographics (from methods above), and analyzed using two‐sided Wilcoxon signed‐rank tests (Lovelace & Brickman, 2013).

## 3 RESULTS

### 3.1 Demographic composition of ecology students

**FIGURE 1:**
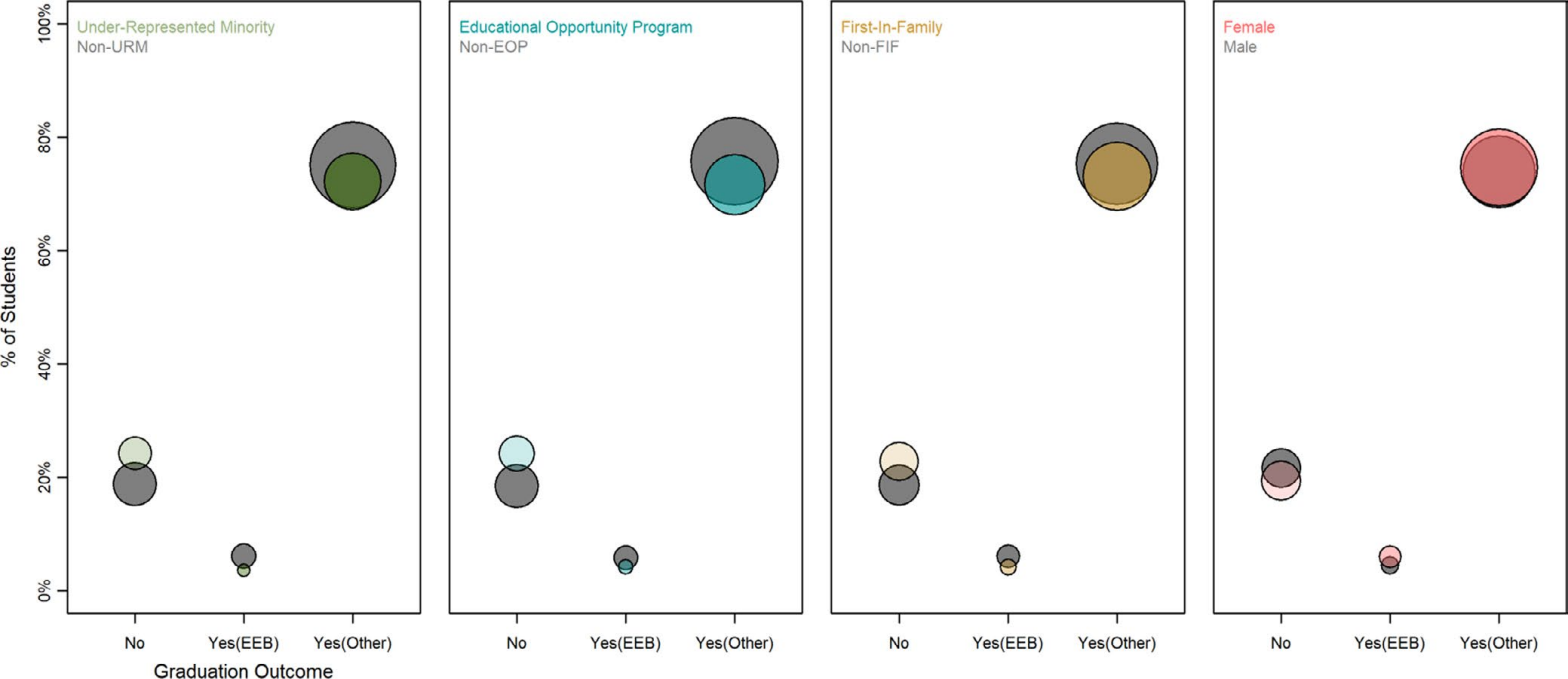
Gaps in 5‐year college completion and EEB retention for all students admitted to UC Santa Cruz between 2008 and 2014 (*N* = 28,500), split by demographic group (panels). Dots are scaled by sample size ((√N)*0.1)

**FIGURE 2:**
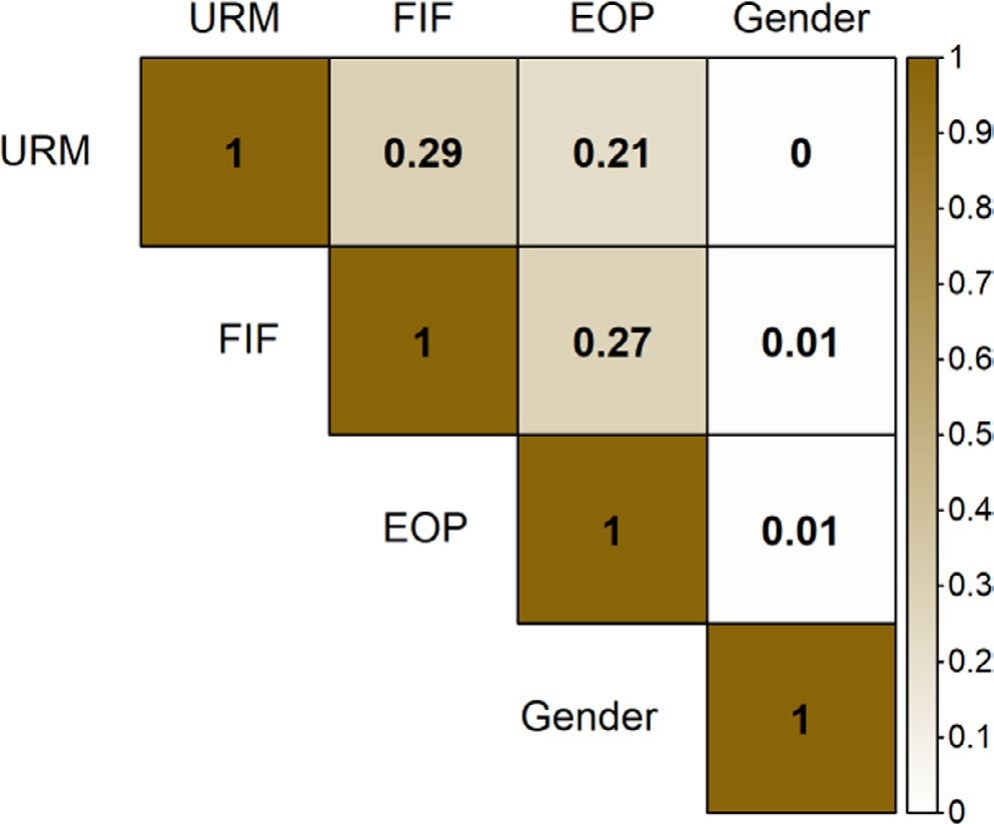
Pairwise correlations between under‐represented groups: under‐represented minority (URM), first‐in‐family status (FIF), socioeconomic status (Educational Opportunity Program status, EOP), and gender. Values are measured as Cramer's V values, scaled between 0 (no association, white) and 1 (full association, brown)

We evaluated admission, enrollment, and graduation trends for UC Santa Cruz (UCSC) students belonging to four groups traditionally under‐represented in higher education: racial/ethnic minority, first‐generation college‐bound, low socio‐economic status, and women students. Upon admission to the university, students from each demographic group were admitted equally as undeclared, EEB, or non‐EEB majors (χ^2^, *p* > .05 for all). However, five years after admission, all under‐represented demographic groups except women were significantly less likely to graduate with EEB degrees (Figure 1). This lower persistence of under‐represented students in EEB suggests an issue with retention rather than attraction to the majors. This retention gap is partly due to a lower rate of college completion and partly through attrition to other majors (Figure 1). Specifically, for students that entered UCSC between 2004 and 2012 with a declared major in EEB, under‐represented groups were significantly less likely to graduate with an EEB major (URM −16%, FIF −15%, EOP −14%) than their peers, being both less likely to complete college (URM −12%, FIF −7%, EOP −10%) and more likely to graduate with a non‐EEB degree (URM + 4%, FIF + 8%, EOP + 5%). Women and men were equally likely to graduate with a degree in EEB. Similarly, students admitted as undeclared majors or declared non‐EEB majors exhibited a persistence gap between under‐represented students and their peers ranging from 1%–2%, of whom 3%–7% did not graduate and 1%–7% graduated in majors other than EEB. For students who graduated in five years with an EEB degree, there was a disparity in graduation grade point average between under‐represented students and their peers. This achievement gap was −0.13 grade points for under‐represented minority students, −0.16 for low socio‐economic students, and −0.15 for first‐generation students. Women had a 0.08 higher GPA than their peers. All demographic groups except gender were significantly and positively correlated with one another, suggesting that intersecting identities play an important role in academic dynamics (Figure 2).

### 3.2 Demographic composition of ecology field courses

**FIGURE 3:**
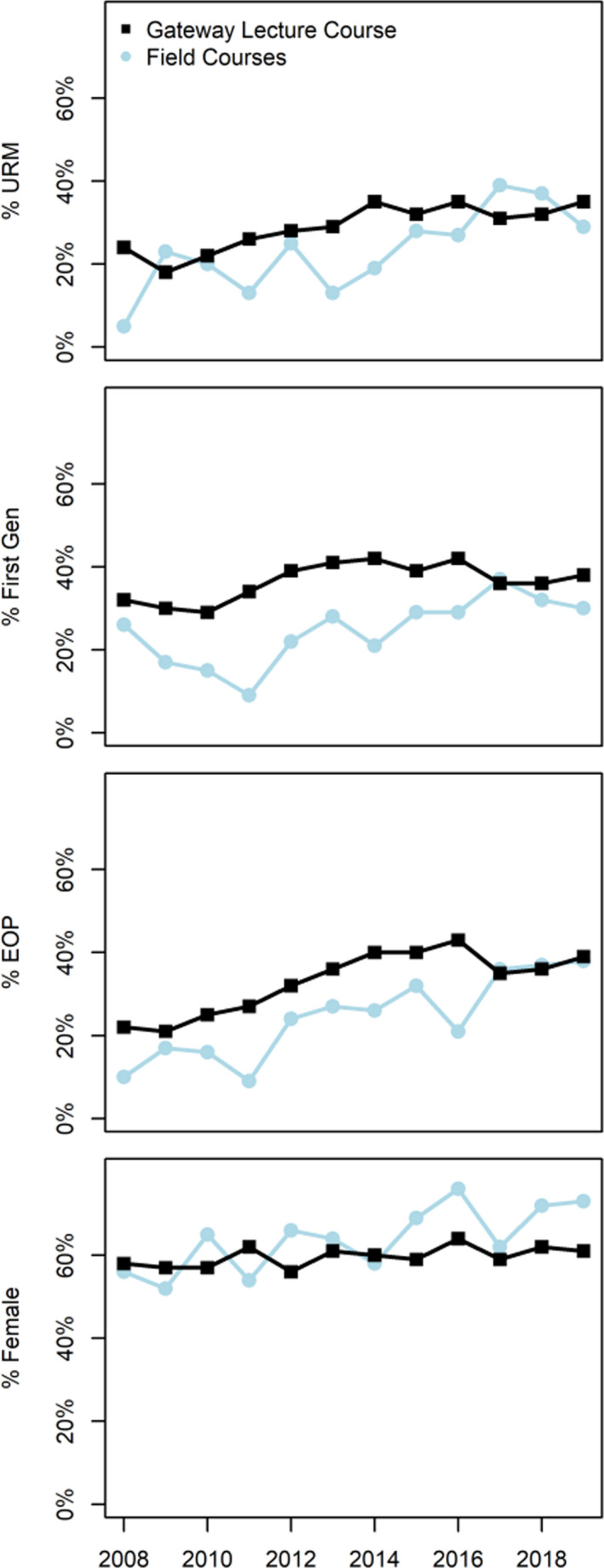
Demographic gaps in enrollment between the gateway biology lecture course BIOE20C (*N* = 11,589 student enrollments, black squares) and the UCSC Ecology and Evolutionary Biology field courses (*N* = 1,239 student enrollments in six courses, blue circles)

Given that field courses are an elective opportunity for students at UCSC (i.e., not required to complete any EEB majors), we next evaluated the demographic composition of ecology field courses since 2008. Of students who graduated within five years as EEB majors (*N* = 1,505), 246 (16%) took at least one field course and 1,259 did not (84%). These enrollments were not distributed evenly across demographic groups: Under‐represented minority students were 2% less likely, low socioeconomic students were 4% less likely, and first‐generation students were 6% less likely than their peers to enroll in field courses. Women were 2% more likely than men to enroll in field courses. Consequently, the demographic composition of field courses over the past 11 years has remained below the diversity rates of the gateway biology lecture course that is required for all EEB majors (Figure 3). On average, the diversity gap in field course participation (calculated as Field Course Diversity ‐ Lecture Course Diversity) was −6% for under‐represented minority students, −9% for low socio‐economic students, and −12% for first‐generation students. The maximum annual diversity gap across this period was −19% for under‐represented minority students, −22% for low socio‐economic students, and −25% for first‐generation students. The diversity gap for women was + 4% on average. While progress has been made and representation of marginalized students has increased over the past three years, the diversity gap in field courses remains (Figure 3).

### 3.3 Links between field courses and student retention, academic success, and career trajectories

Regardless of their declared major at admission, UCSC students who enrolled in at least one field course were more likely to graduate in five years as an EEB major (Table 1). Of students admitted as undecided majors, those who took field courses had a 76% chance of graduating as an EEB major relative to 4% for those who did not take a field course. Similarly, of students admitted as non‐EEB majors, those who took field courses had an 84% chance of graduating as an EEB major relative to 3% for those who did not. Field courses were also associated with retention for students admitted as EEB majors. Specifically, those who took at least one field course were more likely to graduate in EEB (90%) than those who did not take a field course (27%).

**TABLE 1:** Contingency tables showing the proportion of students that graduated in five years as ecology and evolutionary biology (EEB) majors, graduated in five years as non‐EEB majors, or did not graduate based on their major proposed at admission and whether they enrolled in a field course

	Enrolled in Field Course	
	Yes	No
Admitted as undecided majors (χ^2^ = 303.99, odds ratio = 55.84 *df* = 2, *p *< .0001)		
Graduated EEB	19 (76%)	148 (4%)
Graduated Non‐EEB	6 (24%)	2,669 (71%)
Did Not Graduate	0 (0%)	916 (25%)
TOTAL	25	3,733
Admitted as non‐EEB majors (χ^2^ = 2,351.3, odds ratio = 170.35, *df* = 2, *p *< .0001)		
Graduated EEB	88 (84%)	597 (3%)
Graduated Non‐EEB	15 (14%)	17,514 (78%)
Did Not Graduate	2 (2%)	4,469 (20%)
TOTAL	105	22,580
Admitted as EEB majors (χ^2^ = 260.02, odds ratio = 18.58, *df* = 2, *p *< .0001)		
Graduated EEB	139 (90%)	514 (27%)
Graduated Non‐EEB	14 (9%)	973 (51%)
Did Not Graduate	2 (1%)	415 (22%)
TOTAL	155	1,902

Note

Values are given as sample size (proportion of row total).

For those who graduated within five years, student demographics and field course enrollment were both important in predicting GPA at graduation (Table 2). The significant factors appearing in the final model included major at admission and graduation, under‐represented status (negative effect), and field course enrollment (positive effect). Each of the three steps added significantly to the regression model analysis of academic success. The final model accounted for 8.1% of the variance in academic success. Of those who graduated with EEB degrees, students who took at least one field course had significantly higher GPAs than those who did not (3.35 vs. 3.05, t_402_ = −12.146, *p* < .0001, *N* = 246 and 1,259). For field course enrollees, the achievement gap between under‐represented students and their peers was significantly lower (URM: −0.08 for field course enrollees vs. −0.17 for non‐field course enrollees; EOP + 0.05 vs. −0.21; FIF −0.07 vs. −0.18). For students who graduated with non‐EEB degrees, students who took at least one field course had marginally higher GPAs (3.34 vs. 3.21, t_34_ = −1.9308, *p* = .06). There were no significant differences in GPA at graduation among students who enrolled in one, two, or three field courses.

**TABLE 2:** Linear model fits for graduation grade point average as a function of academic, demographic, and field course enrollment variables. Parameter estimates are provided as unstandardized (B) and standardized (β) coefficients

Variable	Model 1			Model 2			Model 3		
	B ± SE	β	t	B ± SE	β	t	B ± SE	β	t
Intercept	3.09 ± 0.01	0.00	253.03***	3.19 ± 0.01	0.00	256.23***	3.15 ± 0.01	0.00	235.86***
Grad Major – non‐EEB	0.09 ± 0.01	0.60	8.01***	0.12 ± 0.01	0.08	10.57***	0.16 ± 0.01	0.10	13.03***
Admit Major – non‐EEB	0.06 ± 0.01	0.06	4.90***	0.05 ± 0.01	0.05	4.28***	0.06 ± 0.01	0.06	5.08***
Admit Major ‐ undeclared	0.03 ± 0.01	0.03	2.63***	0.03 ± 0.01	0.02	2.25*	0.04 ± 0.01	0.03	2.92**
First‐in‐family				−0.08 ± 0.01	−0.12	−9.96***	−0.08 ± 0.01	−0.11	−9.87***
Educational Opportunity Program				−0.06 ± 0.01	−0.11	−5.75***	−0.06 ± 0.01	−0.11	−5.81***
Under‐represented minority				−0.08 ± 0.01	−0.09	−11.89***	−0.08 ± 0.01	−0.09	−11.89***
Men				0.10 ± 0.01	−0.12	18.35***	0.10 ± 0.01	−0.12	18.25***
Field course enrollment							0.24 ± 0.03	0.07	9.48***
**R^2^**	**0.0065**			**0.0767**			**0.0808**		

Note

Stars denote significance.

* *p* < .05;

** *p* < .01;

*** *p* < .001.

### 3.4 Self‐efficacy gains during field courses as an explanatory mechanism

**FIGURE 4:**
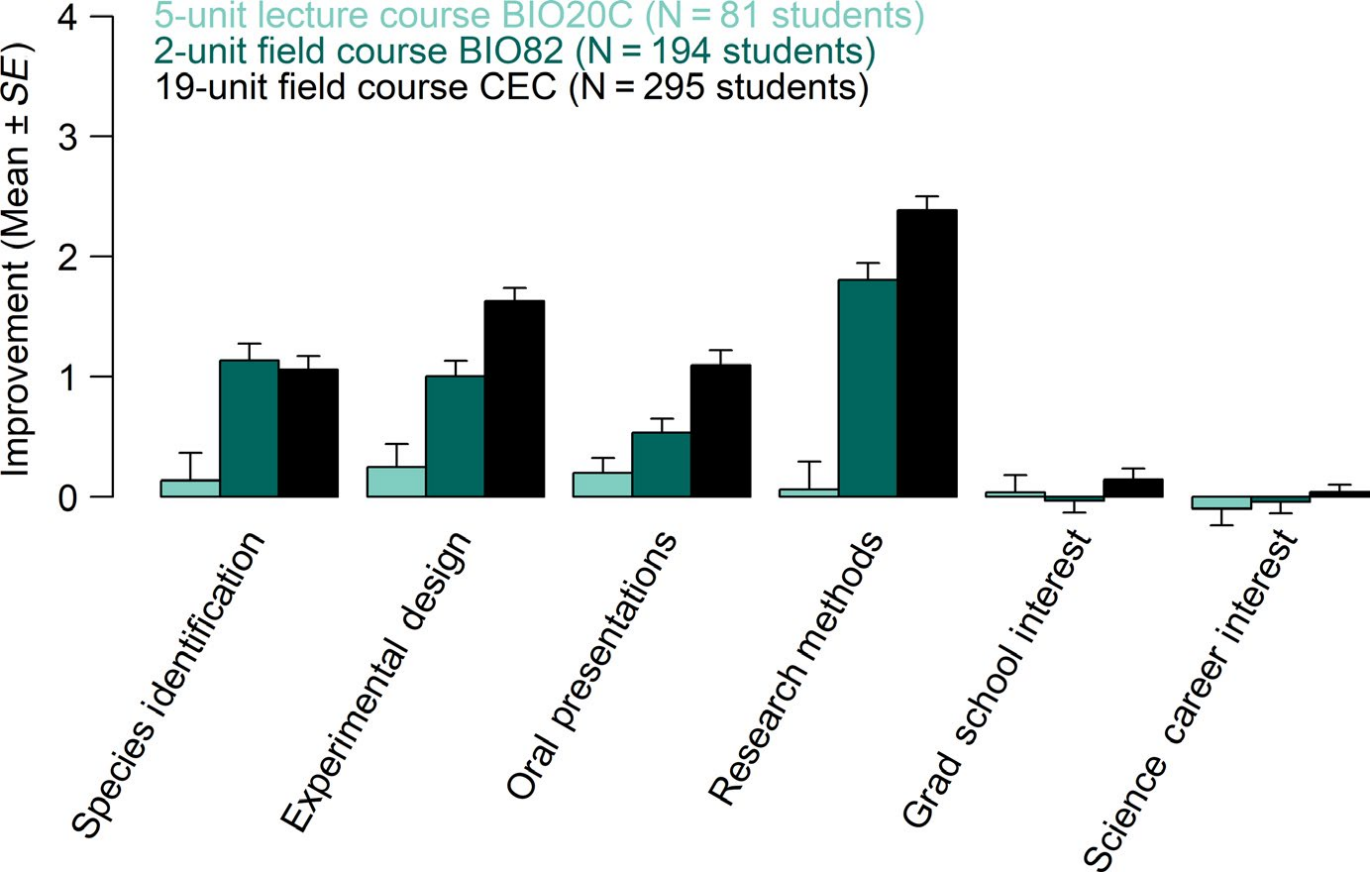
Field courses increase student self‐efficacy more than a lecture course. Improvement values are the difference in self‐ranked confidence between pre‐ and postsurveys, across all demographic groups

Next, we tested whether self‐efficacy gains from field courses could help explain the benefits of field courses. Students enrolled in the gateway lecture course BIO20C did not experience significant increases in self‐efficacy (e.g., self‐reported confidence in scientific skills including oral presentations, species identification) during the course (*p* > .05 for each, Figure 4). In this course, self‐efficacy gains were independent of race/ethnicity and first‐in‐family status, and low socio‐economic students were less likely to desire a science career after the lecture class (*p* = .02886).

**FIGURE 5:**
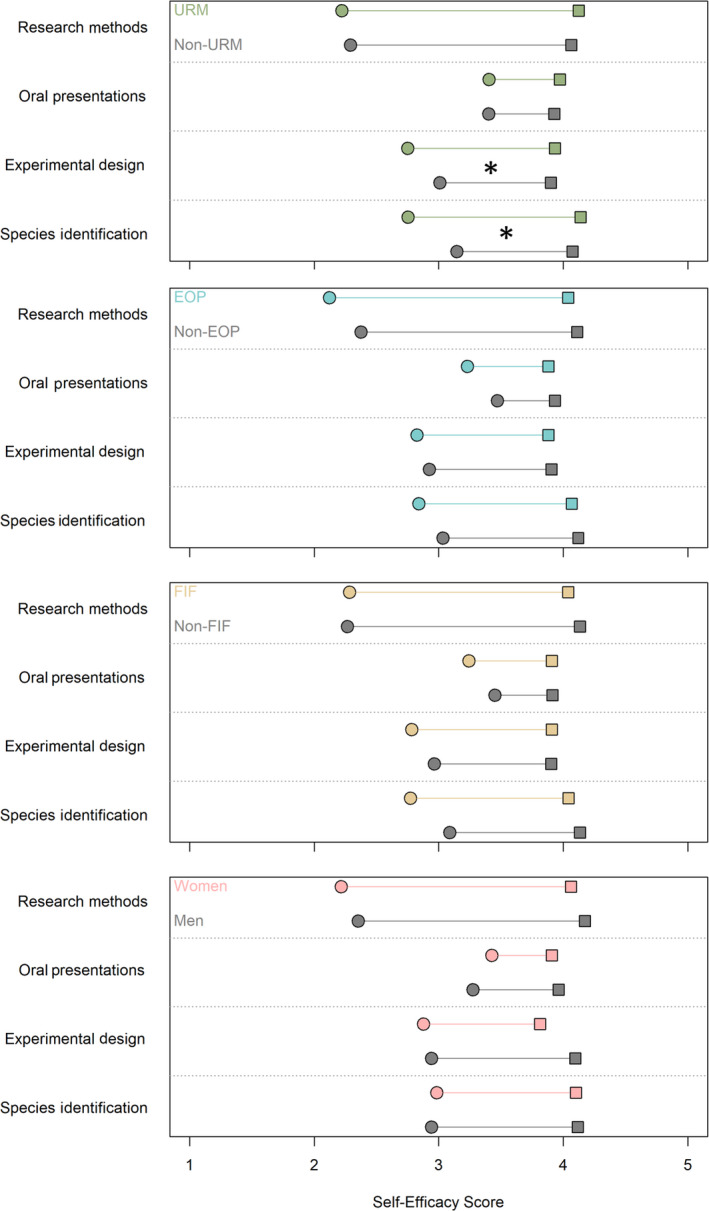
Pre‐ (circles) and post‐ (squares) self‐efficacy scores for under‐represented minority (URM, green), economically disadvantaged (EOP, blue), first‐generation (FIF, yellow), and women (pink) students in the lower‐division field course BIO82 (*N* = 194). Starred pairs of pre–post gains differed significantly (*p* < .05)

Students enrolled in the lower‐division field course BIO82 reported increased confidence in their ability to identify flora/fauna species, design experiments, deliver oral presentations, employ field research methods, and obtain further hands‐on opportunities at UCSC (*p* < .0001 for each, Figure 4). In this course, race/ethnicity was the only demographic factor to influence self‐efficacy (Figure 5). Under‐represented minority students began the course with lower confidence than their peers in their ability to identify California flora and fauna and design experiments, but gained more confidence during the course than non‐URM students (*p* = .002894 and *p* = .02667, respectively) and thus ended the course with comparable confidence. First‐in‐family students began the course with significantly higher interest in pursuing a graduate degree (*p* = .0349). There was no effect of socioeconomic status or gender on self‐efficacy scores before or after the course.

The upper‐division field course CEC had a particularly strong influence on student self‐efficacy as compared to the lower‐division field course BIO82, with significantly higher gains in all categories except identification of flora and fauna (Figure 4). Neither the lecture course nor the field courses increased student interest in attending graduate school or working toward a career in science, likely because the pre‐course values were already high across all courses (raw score out of 5 ranged 4.1 to 4.4 for Grad Degree, and 4.4 to 4.7 for Science Career, Appendix Table S2). In the CEC course, race/ethnicity and first‐generation status both affected self‐efficacy gains (Figure 5). Under‐represented minority students began the course with lower confidence in their ability to identify California flora and fauna, but gained more confidence during the course than non‐URM students (*p* = .003187) and thus ended the course with comparable confidence. Under‐represented minority students began the course with lower interest in a science career and gained marginally more interest than their peers (*p* = .06329). First‐in‐family students began the course with the same interest in a science career as their peers but gained significantly more during the course (*p* = .01217).

## 4 DISCUSSION

We found that field courses increased self‐efficacy and narrowed the achievement and completion gap in marginalized demographic groups (Figure 4, Table 1, Table 2). Compared to the 5‐unit lecture course, field courses had a disproportionately large and positive impact on self‐assessment of core science competencies (hereafter, self‐efficacy) (Figure 4). We found that a lower‐division field course provided a low‐cost option that still conferred significant self‐efficacy advantages (Figure 4), engaged students early in their career (Toven‐Lindsey et al., 2015), and required less class time and instruction than the lecture course (Appendix Table S1). In comparison, the upper‐division field course provided a more intensive option that resulted in twofold higher self‐efficacy advantages, but was more costly for students and the campus (Figure 4, Appendix Table S1). Both field courses closed gaps in self‐efficacy between under‐represented students and their peers (Figure 5) while the lecture course did not, indicating that field courses can be an important vehicle for inclusion and equity in EEB. Together, these data suggest that field courses may provide a promising pathway for diversifying the STEM workforce.

These findings are especially promising given that Ecology and Evolutionary Biology (EEB) appears to have a retention rather than a recruitment problem for diverse undergraduate students (Figure 1). Specifically, we found that students from under‐represented groups were equally likely to be admitted as proposed EEB majors and have high science career and graduate school interest (Figure 4). However, students from under‐represented groups had a lower rate of college completion and higher rate of attrition to other majors (Figure 1). Additionally, under‐represented students that graduated with EEB degrees had a GPA deficit compared to their peers (Table 2). Our findings align with previous studies showing that URM students entering college are just as likely as their non‐URM peers to intend pursuit of a scientific career but over the course of undergraduate studies, these students are disproportionately lost (Koenig, 2009; Toven‐Lindsey et al., 2015).

We also discovered that field courses had low URM representation, although this gap appears to be closing at our focal institution (Figure 3). Field courses are often over‐subscribed, with student demand exceeding enrollment supply. However, the enrollment selection process at UCSC does not depend on grade point average and thus does not select for high‐achieving students. Additionally, the selection process does not consider race or ethnicity and thus cannot explain the disparate enrollment rates of students from marginalized groups. Instead, we suggest that course fees, cost of field equipment such as camping gear, a potential perception of lacking necessary experience to be competitive, and specialized training may serve as barriers to participation. In our study, courses with large fees (up to $3,000) and/or requirements for specialized equipment and certifications (e.g. SCUBA), expensive or complicated travel requirements, tended to include fewer socio‐economically disadvantaged and first‐generation students than other field courses (Appendix Table S1). Anecdotal data from participants in BIOE82, which has a course fee of $180, suggest that even a moderate fee is enough to deter students from enrolling and likely presents enough of a barrier to keep some students from applying. To our knowledge, this study is the first to compare the participation of under‐represented groups in costly elective courses within a single university. In the future, we suggest that the demographics of students should be tracked through the field courses application process to check for equity in access (Tanner, 2009). Future research should also seek to quantify this enrollment disparity across institutions and nations with a spectrum of education costs.

Given their effectiveness at enhancing graduation rates and GPAs for all students, and for closing achievement gaps in under‐represented students, increasing field course opportunities and eliminating enrollment barriers must be a priority. Specifically, increased opportunities for early field course enrollment, especially in the lower‐division field course with a minimal course fee (Appendix Table S1), can address broader disparities in STEM major completion and college graduation rates. This can be facilitated by increasing the number of course offerings, providing course fee scholarships, or adding field courses to major requirements. A more inclusive application system would also institutionalize the advancement of diversity instead of relying on faculty advocates from marginalized groups (Jimenez et al., 2019) or with education specialties (Bush et al., 2008).

The number of field course offerings for undergraduates is limited for a variety of reasons. Instructors cite time commitments and content forfeiture as impediments (Brownell & Tanner, 2012; Jimenez et al., 2019); however, recent evidence demonstrates that field courses facilitate significant learning of fundamental concepts (Elkins & Elkins, 2007), and institutions are increasingly incentivizing teaching approaches that promote diversity (Edwards & Roy, 2017). Thus, field courses need not create a trade‐off between field‐based experiences and the content goals of a given curriculum. More rigorous evaluation of field courses requiring varying time‐commitments, equipment, and fees is necessary to identify the most effective and scalable approaches for promoting increased institutional adoption of field courses (Ballen & Mason, 2017; Graham et al., 2013; Mervis, 2006). A cost‐benefit analysis of institutional investment in various types of courses has yet to be completed, and debate between resource allocation to single large or several small classes remains. In the future, these studies will be critical for a data‐driven approach to informing curriculum and academic plan development.

There are several potentially confounding factors related to field courses and student outcomes, including: self‐selection of more successful students into field courses, differences in grades between field and lecture courses, and smaller class sizes of students. However, lower‐division field course (BIO82) students began the course with confidence in their science‐specific skills (e.g., oral presentations, species identification) comparable to the lower‐division lecture course (BIO20C) students suggesting that student success bias is not the case (Appendix Table S2). Further, at the upper‐division level, the grade allocations of field courses are distributed similarly to those of lecture courses. Additionally, previous studies have shown that the small class size of field courses is unlikely to drive trends in student success because upper‐division lecture courses are of comparable size (Ballen et al., 2018). Regardless, we can say with certainty that field courses were associated with higher retention and success (Tables 1 and 2). Finally, we note that institutional context can impact student perceptions and experiences and suggest that similar studies be undertaken at other universities including community colleges and liberal arts colleges.

Future research efforts should seek to understand how social (Estrada, Woodcock, Hernandez, & Schultz, 2011; Torregosa, Ynalvez, & Morin, 2016) and psychological (Hanauer, Graham, & Hatfull, 2016) mechanisms such as sense of belonging, project ownership, and community building explain the benefits of field courses. Like Rainey, Dancy, Mickelson, Stearns, and Moller (2018), we found that students from under‐represented groups have lower self‐efficacy in the sciences (Browman, Destin, Kearney, & Levine, 2019). Due to logistical constraints, we did not account for hidden identities such as sexual orientation, political affiliation, and religion that likely play an important role (Henning, Ballen, Molina, & Cotner, 2019). A qualitative research approach including focus groups and reflective journaling would help to evaluate other roadblocks to entry such as family planning considerations (Lynn, Howells, & Stein, 2018) and sexual harassment (Leaper & Starr, 2019). Understanding barriers perceived by students can inform targeted interventions to reinforce the academic pipeline (Freeman, Landry, Trevino, Grande, & Shea, 2016). Additionally, the degree to which field course benefits can be attributed to pedagogical methods employed in a course (e.g., active learning) or context in which a course is taught (e.g., in nature) must be studied in the future. Finally, we recognize the limitations of combining all under‐represented minority groups into a single category. In the future, an in‐depth analysis of experiences within and between race/ethnicity groups would provide valuable insight.

In addition to reforming academic practice, we must work toward inclusion and equity across all levels. What was previously termed the “leaky pipeline” is now understood to include biases across critical STEM processes (Grogan, 2019) including publishing (Bendels, Müller, Brueggmann, & Groneberg, 2018; Holman et al., 2018; Murray, Siler, Lariviére, et al., 2019; Murray, Siler, Larivière, et al., 2019), funding (Ginther et al., 2011; Hechtman et al., 2018; Sege, Nykiel‐Bub, & Selk, 2015; Van der Lee & Ellemers, 2015), hiring (Eaton et al., 2019; Moss‐Racusin, Dovidio, Brescoll, Graham, & Handelsman, 2012; Sheltzer & Smith, 2014), promotion (Kaminski & Geisler, 2012; Lerchenmueller & Sorenson, 2018; Van Dijk, Manor, & Carey, 2014), recommendation (Dutt, Pfaff, Bernstein, Dillard, & Block, 2016; Schmader, Whitehead, & Wysocki, 2007), and recognition (Lincoln, Pincus, Koster, & Leboy, 2012; Ma, Oliveira, Woodruff, & Uzzi, 2019). Decades of directed effort have somewhat lessened these biases and led to more institutional representation of women scientists (Cheryan et al., 2017), with academia predicted to reach gender parity in 50 years (Holman et al., 2018). Yet these biases add to existing academic performance barriers faced by students whose parents complete fewer years of education or have less financial resources (Ma, Pender, & Welch, 2016; Sirin, 2005). As a result, the progress for other groups such as under‐represented minorities and low socio‐economic students is markedly slower. Worldwide, gender and racial gaps in STEM representation remain (Holman et al., 2018), and disparities worsen at higher levels of academia. For example, UCSC undergraduate students are 53% women compared to 45% of graduate students, 39% of postdoctoral researchers, and 42% of tenure‐track faculty at the same institution. In contrast, the racial diversity gap is far more severe, as undergraduates are 38% URM, compared to 18% of graduate students, 15% of postdocs, and 11% of ladder‐rank faculty. Ecology and Evolutionary Biology in particular lags other STEM fields, including biomedical, cellular, and molecular biology (5.8% URM graduate students in EEB vs. 10.1% URM) across the United States (NSF, 2015).

We must continue to identify practical and scalable steps to promote inclusion and success of students from historically marginalized populations (Ballen et al., 2018). The resulting “diversity dividend” will benefit scientific quality and progress (AlShebli, Rahwan, & Woon, 2018; Campbell, Mehtani, Dozier, & Rinehart, 2013; Nielsen et al., 2017). Additionally, diverse leaders can inspire diverse students, leading to a positive feedback loop that can have lasting impacts (Hernandez et al., 2018; Potvin, Burdfield‐Steel, Potvin, & Heap, 2018; Price, 2010). Thus, broadening participation and achievement of racial minorities in STEM must continue to be an important community goal (Canning et al., 2019; Nielsen et al., 2017).

## CONFLICT OF INTEREST

The authors declare no competing interests.

## AUTHOR CONTRIBUTION

**Roxanne Beltran:** Conceptualization (equal); Data curation (equal); Formal analysis (equal); Methodology (equal); Project administration (equal); Visualization (equal); Writing‐original draft (equal); Writing‐review & editing (equal). **Erin Marnocha:** Data curation (equal); Formal analysis (equal); Methodology (equal); Project administration (equal); Writing‐review & editing (equal). **Alexandra Race:** Data curation (equal); Methodology (equal); Validation (equal); Writing‐review & editing (equal). **Don Croll:** Data curation (equal); Investigation (equal); Project administration (equal); Writing‐review & editing (equal). **Gage Dayton:** Data curation (equal); Funding acquisition (equal); Project administration (equal); Writing‐review & editing (equal). **Erika Zavaleta:** Conceptualization (equal); Data curation (equal); Formal analysis (equal); Funding acquisition (equal); Project administration (equal); Writing‐original draft (equal); Writing‐review & editing (equal).

## Supporting information

Supplementary Material

## Data Availability

Aggregate data are archived in the publicly accessible repository Dryad at https://doi.org/10.7291/D1DM3P.
