# Supplementary material for: Field courses narrow demographic achievement gaps in ecology and evolutionary biology
Source: Ecol Evol. 2020 May 8;10(12):5184–96. doi: 10.1002/ece3.6300 (PMC7319162; doi:10.1002/ece3.6300)
Supplement: Supplementary file 1 — Supplementary Material [file ECE3-10-5184-s001.docx]

**Appendix Table 1.** Descriptive information about the lecture and field courses included in the demographic analysis: the number of students enrolled from 2008-2019, the approximate course fee required for enrollment in addition to tuition, whether the course required a SCUBA certification, and the proportion of Educational Opportunity Program (EOP), under-represented minority (URM), and first-in-family (FIF) students enrolled.

| **Course Number** | **Course Name** | **# Students** | **Course Fee** | **SCUBA** | **% EOP** | **% URM** | **% FIF** |
| --- | --- | --- | --- | --- | --- | --- | --- |
| BIOE 20C (lecture) | Ecology and Evolution | 11,589 | $0 | N | 33% | 29% | 37% |
| BIOE 82 (field) | Intro to Field Research & Conservation | 411 | $180 | N | 42% | 41% | 44% |
| BIOE 128L (field) | Large Marine Vertebrates | 113 | $150 | N | 22% | 19% | 27% |
| BIOE 151 (field) | Ecology & Conservation in Practice | 96 | $1,600 | N | 32% | 21% | 28% |
| BIOE 159 (field) | Marine Ecology Field Quarter | 223 | $3,000 | Y/N | 23% | 19% | 25% |
| BIOE 161 (field) | Kelp Forest Ecology | 146 | $350 | Y | 14% | 14% | 16% |
| BIOE 75 (field) | Scientific Diving | 250 | $3,000 | Y | 14% | 20% | 18% |

**Appendix Table 2.** Mean survey scores from students in the 5-unit lecture course BIO20C, 2-unit field course BIO82, and 19-unit field course CEC. Pre- and post-values are given on a 5-point Likert scale (1=strongly disagree, 2=disagree, 3=neither, 4=agree, 5=strongly agree) and change values are given as (post-pre).

|  | **5-unit lecture BIO20C** | | | **2-unit field BIO82** | | | **19-unit field CEC** | | |
| --- | --- | --- | --- | --- | --- | --- | --- | --- | --- |
| **Category** | **Pre** | **Post** | **Change** | **Pre** | **Post** | **Change** | **Pre** | **Post** | **Change** |
| Experimental Design | 3.30 | 3.40 | +0.14 | 2.96 | 4.10 | +1.14 | 3.28 | 4.34 | +1.06 |
| Research Methods | 3.17 | 3.42 | +0.25 | 2.88 | 3.90 | +1.01 | 2.83 | 4.47 | +1.63 |
| Species Identification | 3.21 | 3.41 | +0.20 | 3.38 | 3.91 | +0.54 | 3.25 | 4.36 | +1.10 |
| Oral Presentation | 2.51 | 2.57 | +0.06 | 2.28 | 4.08 | +1.81 | 2.32 | 4.71 | +2.38 |
| Grad School Interest | 4.35 | 4.33 | +0.04 | 4.09 | 4.06 | -0.03 | 4.09 | 4.25 | +0.14 |
| Science Career Interest | 4.48 | 4.38 | -0.10 | 4.43 | 4.38 | -0.04 | 4.68 | 4.72 | +0.04 |
